# *Mycoplasma synoviae* Induces Apoptosis in Chicken Oviduct Cells

**DOI:** 10.3390/vetsci11120639

**Published:** 2024-12-10

**Authors:** Xudong Zhang, Xiaochun Wu, Yuting Zhang, Yulu Chen, Tingwen Li, Yuan Shi, Shijun Bao

**Affiliations:** College of Veterinary Medicine, Gansu Agricultural University, Lanzhou 730070, China; zxd82598@163.com (X.Z.); zyt070998@163.com (Y.Z.); cylsgxka520@163.com (Y.C.); 18893530763@163.com (T.L.); syuanhi@163.com (Y.S.)

**Keywords:** *Mycoplasma synoviae*, chicken oviduct cells, proliferation, apoptosis

## Abstract

*Mycoplasma synoviae* (MS) is an important pathogen that can cause respiratory diseases in poultry and birds, leading to serious economic losses in the world and impacting the development of the poultry industry. However, the pathogenic mechanisms of MS infection are still unclear, resulting in a lack of effective diagnosis and treatment strategies. This study aimed to uncover the infection effect caused by MS in chicken oviduct cells. MS-infected chicken oviduct cells with different infection times and doses were collected, and DAPI staining was performed to monitor the morphological changes of cell nuclei. Cell viability was detect by CCK-8 assay. The expression of marker genes of cell apoptosis was determined by RT-qPCR and Western blotting assay, respectively. The results showed that MS infection significantly inhibited the growth of chicken oviduct cells, caused the nuclear shrinkage and rupture, activated the expression of apoptosis marker genes Caspase 3 and Beclin-1, and increased the apoptosis rate. These results indicated that MS infection inhibits cell proliferation and promotes apoptosis in chicken oviduct cells, which provided a basis for further revealing the MS pathogenic mechanism and provided a foundation for the future development of anti-infection strategies.

## Simple Summary

*Mycoplasma synoviae* is an important pathogen that poses a serious threat to the poultry industry and causes serious economic damage worldwide. However, there is limited knowledge about the pathogenic mechanism of *Mycoplasma synoviae* infection, resulting in a lack of effective diagnosis and treatment strategies. In this study, we found that *Mycoplasma synoviae* infection resulted in morphological changes in chicken oviduct cells, nucleus crumpling and fragmentation, inhibition of cell viability, and promotion of apoptosis, which provided a basis for further research revealing the infection mechanism caused by *Mycoplasma synoviae*.

## 1. Introduction

*Mycoplasma* is a pleomorphic bacterium with self-replicating ability, lacking a cell wall, and having a very low GC content in its genome [1,2]. *Mycoplasma synoviae* (MS) is a common poultry pathogen that can cause upper respiratory disease, infectious synovitis, arthritis, eggshell apex abnormalities, and decreased egg production, growth, and hatchability in chickens [3,4]. MS infection compromises the immune system of chickens. And the symptoms caused by MS infection are difficult to distinguish from those caused by other avian pathogens, which lead to increased mortality in poultry [5,6]. Many samples suspected to be infected with MS were reportedly collected from different provinces in China and showed that the MS positivity rate was 66.53% [7], which caused huge annual economic losses to the poultry industry globally [8].

Most mycoplasmas exhibit strict host specificity but limited biosynthetic ability. Due to the lack of a cell wall, mycoplasma adhesins, an integral part of the membrane, enable mycoplasma to bind to specific receptors on the host cell membrane, which can allow the mycoplasma to uptake important nutrients and metabolite accumulation, ultimately damaging the host cell membrane [9]. Currently, MS exhibits resistance to existing antibiotics. Although some subunit and inactivated vaccines have been developed to prevent MS, they are still insufficient to control the widespread impact of this disease [10,11]. The development of novel and effective prevention and treatment strategies remains an urgent problem to be solved. However, knowledge about the infection and pathogenic mechanism of MS is still limited. Revealing the MS infection mechanism will provide a foundation for the development of prevention and treatment strategies for diseases [12].

Apoptosis is a form of programmed cell death, playing an important role in embryonic development, tissue repair, and homeostasis of the internal environment of the organism [13]. Studies have revealed that some *Mycoplasma* strain infections induce apoptosis in host cells [14]. Recent research reported that MS infection stimulates apoptosis-related factors in chicken synovial fibroblasts [2]. However, the mechanism by which MS causes decreased egg production quality remains unknown. This study attempted to construct MS-infected chicken oviduct cells and explore the MS-induced infection mechanism in chicken oviduct cells, which would provide a theoretical basis for the development of prevention and treatment strategies for MS.

## 2. Materials and Methods

### 2.1. Identification of MS and Titer Determination

The MS strain was isolated and kept in the Laboratory of Infectious Diseases, Gansu Agricultural University (Lanzhou, China). The strain was identified via detecting 16sRNA using PCR assay. The primers listed as follows were generated by Tsingke (Beijing, China), and the primer sequences are shown in Table 1. The multiplicity of infection (MOI) of MS was determined by (bacteria titer × bacteria volume)/cell number. The bacteria titer was 3 × 10^9^ TU/mL.

### 2.2. Isolation of Primary Chicken Oviduct Cells

Healthy laying hens aged 23–25 weeks were obtained for the isolation of primary chicken oviduct cells. Briefly, the hens were moved into an ultra-clean bench, and the funneled portion of the chicken oviduct was removed into PBS containing dual antibodies using sterile scissors and forceps. After rinsing repeatedly, the tissues were cut up into small pieces with scissors. Then, 0.2% type II collagenase was added into the tissue culture for 1 h digestion. After the termination of digestion by adding an M199 complete medium, the tissue cultures were filtered via a 200 mesh steel sieve, and the filtrate was collected via centrifuge for 5 min at 1200 rpm. The precipitates were mixed with M199 complete medium and transferred into culture dishes for inoculation. Cells were passaged when the cell density grew to 80%.

### 2.3. Cell Culture and Treatment

Primary chicken oviduct cells were isolated (the name of the ethics committee: Laboratory Animal Ethics Committee of Gansu Agricultural University, Ethical approval code: GSAU-ETH-VMC-2022-014, Date of approval: 14 March 2022) and maintained in M199 (BasalMedia, Shanghai, China) medium containing 10% FBS, 100 IU/mL penicillin, and 100 µg/mL streptomycin (Beyotime, Beijing, China). Before treatment, the cells were digested with trypsin (Grand Island, NY, USA), seeded in 6-well plates (1 × 10^4^ cells/well) and placed in a cell culture incubator for culture (37 °C, 5% CO_2_) [14]. When the cell density reached 70–80%, chicken oviduct cells were incubated with MS at different MOIs (1 × 10^7^ CCU/well) of 20, 50, 80 and 100 and different times after infection of 24 h, 48 h and 72 h, respectively. The cells were then digested with trypsin and collected for the next step of the experiment with each treatment repeated at least three times [15].

### 2.4. Establishment of Immortalized Chicken Oviduct Cell Line

An immortalized chicken oviduct cell line was generated by the lentiviral-mediated hTERT induction method. Briefly, primary chicken oviduct cells were incubated in dishes for 50–70% confluence. Then, a lentiviral packaging plasmid containing hTERT and green fluorescence protein (GFP), generated by Genscript (Nanjing, China), was transfected into cells using a transfection auxiliary reagent GC-LVreinforcer (Genecarer, Xi’an, China) following the manufacturer’s protocol. Green fluorescence can be observed under fluorescence microscopy 48 h after infection. Puromycin was introduced to screen stable cells until the green fluorescence reaches over 80%. The cell line was expanded for cultivation and biological identification. To monitor the cellular characteristics of immortalized oviduct cells, the oval protein expression was detected by RT-qPCR in the 3rd, 7th and 15th generations.

### 2.5. Nuclei Staining

Chicken oviduct cells were cultured into 6-well plates until the cell confluence reaches 70–80%. After infection with 80 MOI of MS for 24 h, 48 h, and 72 h, respectively, chicken oviduct cells were collected and fixed using 4% paraformaldehyde for 15~20 min at room temperature. The cell membrane was penetrated with PBS containing 0.5% Triton-X-100 for 10 min, after which DAPI (Solarbio, Beijing, China) staining solution was added for 15 min at room temperature, and then they were washed with PBS for 5 min each. Finally, the cells were sealed to observe the nuclei morphology by fluorescence microscopy (Echo-Labs, Englewood, CO, USA).

### 2.6. Cell Viability Assay

The cell viability was determined using the CCK-8 method (Vazyme, Nanjing, China) according to the manufacturer’s instruction. In brief, the cells were plated into 96-well plates with 8 × 10^3^ cells per well. After being treated with 20, 50, 80 and 100 MOI of MS at 37 °C for 24 h, 48 h, 72 h, respectively, the cells were maintained in 100 µL of serum-free medium and 100 µL of CCK-8 solution for 2–4 h incubation. Absorbance at 450 nm was measured using an enzyme labeler (Molecular Devices, San Jose, CA, USA).

### 2.7. RT-qPCR Analysis

Total RNA was extracted from chicken oviduct cells using the Trizol method (Beyotime, Shanghai, China). The cDNA was synthesized according to the instructions of the PrimeScript RT Reagent Kit (Takara, Dalian, China) [15]. The primer sequences for the target genes are shown in Table 2. The RT-qPCR was performed using a SYBR Green Master kit (Vazyme, Nanjing, China) following the manufacturer’s instructions. Each sample was repeated three times as technical replicates. Gene mRNA quantification was expressed using the 2^−ΔΔCt^ method.

### 2.8. Western Blotting Analysis

Chicken oviduct cells treated with MS were collected for protein extraction using RIPA lysate with 1% phenylmethylsulfonyl fluoride (PMSF), 1% phosphatase inhibitor, and 1% protease inhibitor (Solarbio, Beijing, China). The protein concentration was determined by a BCA kit (Beyotime, Shanghai, China). Total proteins were separated by 10% sodium dodecyl sulfate-polyacrylamide gel electrophoresis (SDS-PAGE) and electro-transferred to a nitrocellulose filter (NC) membrane (Millipore, Bedford, MA, USA). The NC membrane was closed with 10% skimmed milk powder for 120 min at room temperature and washed three times with PBST for 5 min each time, and then it was incubated overnight at 4 °C with the following primary antibodies: the anti-β-actin antibody (1:10,000, Proteintech, Chicago, IL, USA), anti-caspase3 (ThermoFisher, Waltham, MA, USA), and anti-Beclin antibodies (1:5000, Abcam, Cambridge, UK) [14]. The membrane was washed six times with PBST for five minutes each; then, it was incubated with a rabbit anti-HRP secondary antibody (1:20,000, Bioss, Beijing, China) for 60 min at room temperature and afterwards washed three times with PBST for five minutes each. Blot bands were detected by ECL chemiluminescence reagent (Beyotime, Shanghai, China).

### 2.9. Flow Cytometric Analysis

Flow cytometric was performed to detecting cell apoptosis using an FITC Annexin V Apoptosis Detection Kit (Vazyme, Nanjing, China) according to the manufacturer’s instructions. Data were analyzed by FlowJo software (version 7.6). Briefly, chicken oviduct cells, treated with an 80 MOI of MS in 6-well plates for 24, 48, and 72 h, were digested with EDTA-free trypsin and collected after washing twice using phosphate buffer solution (PBS). Then, the cells were resuspended and incubated with FITC Annexin V and propidium iodide (PI) for 10 min at room temperature. Data were collected by flow cytometry within 1 h.

### 2.10. Statistical Analysis

The data were analyzed using SPSS 19.0 statistical software. The significance of differences between groups was analyzed by a one-way analysis of variance (ANOVA) or two-tailed Student’s *t*-test. A *p*-value of <0.05 was considered to be significant.

## 3. Results

### 3.1. Identification of Mycoplasma synoviae

**Figure 1:**
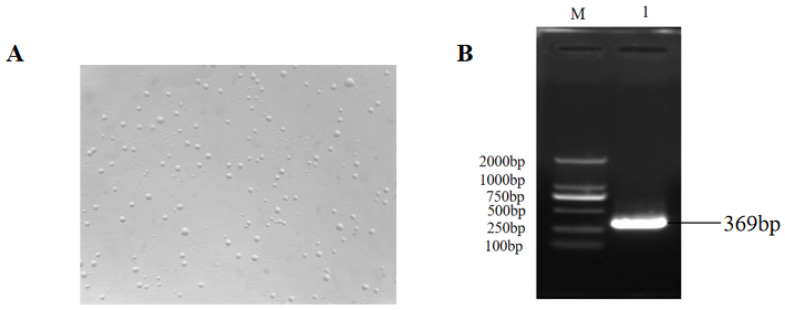
Identification of *Mycoplasma synoviae* (MS). (**A**) The MS colonies on solid Petri dishes. (**B**) Agarose gel electrophoresis verified the specific amplification product of MS with the size of about 369 bp. M: standard molecular marker; 1: PCR amplification product of MS 16sRNA.

To obtain the MS strain for infection, MS preserved in the laboratory was performed for expanding culture and purification by the streaking method on solid Petri dishes. As shown in Figure 1A, after 3–5 days of incubation, the typical “omelette-like” colonies were observed under microscopy. A random single clone was picked for expansion culture, and the genome was extracted for bacterial identification using PCR amplification and 1% agarose electrophoresis. As shown in Figure 1B, the gene band of MS with the size of about 369 bp was obtained, indicating that the MS strain was obtained and can be used for subsequent studies.

### 3.2. Establishment of an Immortalized Chicken Oviduct Cell Line

**Figure 2:**
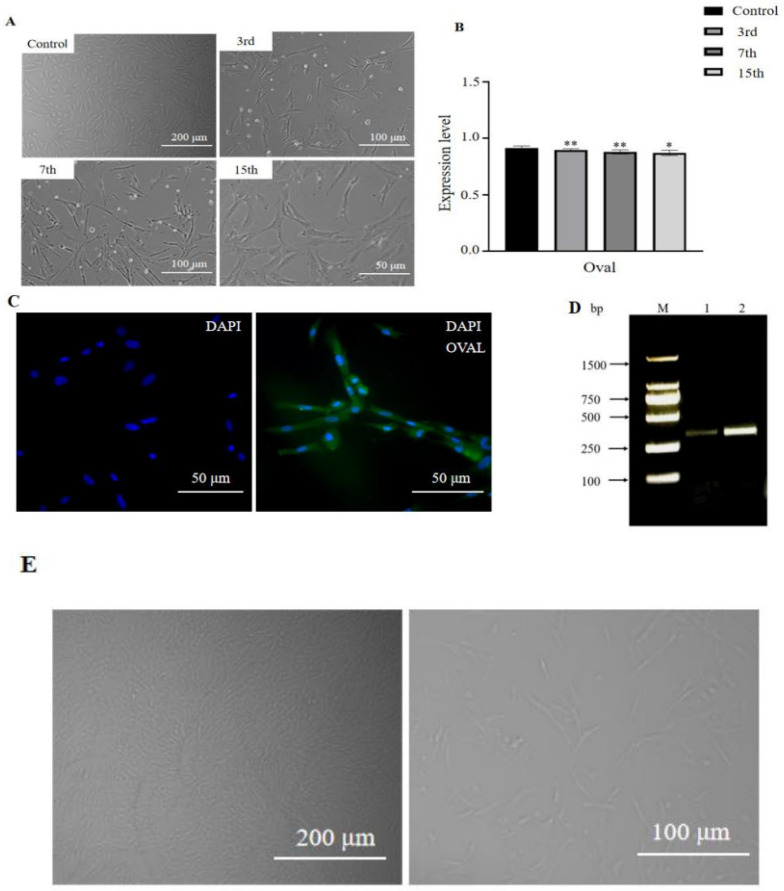
Establishment of immortalized chicken oviduct cell line using lentivirus-mediated exogenous hTERT induction. (**A**) The morphology of primary cells (control) and cell line of different generations after introducing hTERT. (**B**) qPCR detection of the oviduct cell marker gene oval expression in chicken oviduct cell line. (**C**) Identification of positive cells ratio by immunofluorescence. (**D**) PCR was performed to determine the expression of the hTERT gene with a size of about 380 bp in chicken oviduct cells. M: standard molecular marker; 1, 2: hTERT gene fragment. (**E**) shows the 5th and 9th generations of primary cells. Data were analyzed by SPSS, and cell experiments were all repeated three times. * *p* < 0.05, ** *p* < 0.01 (Student’s *t*-test).

To establish the immortalized chicken oviduct cell line, primary chicken oviduct cells were isolated and cultivated from six-month-old hens using the collagenase digestion method. The immortalized chicken oviduct cell line was generated by the lentiviral-mediated exogenous hTERT induction method. As shown in Figure 2, the chicken oviduct cell was continuously passaged to over 15 generations after introducing an exogenous hTERT gene. The cells kept the typical spindle shape as primary cells (Figure 2A). The marker gene oval showed stable expression in the chicken oviduct cell line (Figure 2B). Almost all cells expressed the GFP inherent in lentiviral vectors after puromycin screening (Figure 2C). And the hTERT gene is highly expressed in the cell line (Figure 2D). These results indicated that this study obtained a chicken oviduct cell line.

### 3.3. MS Inhibits the Growth of Chicken Oviduct Cells

**Figure 3:**
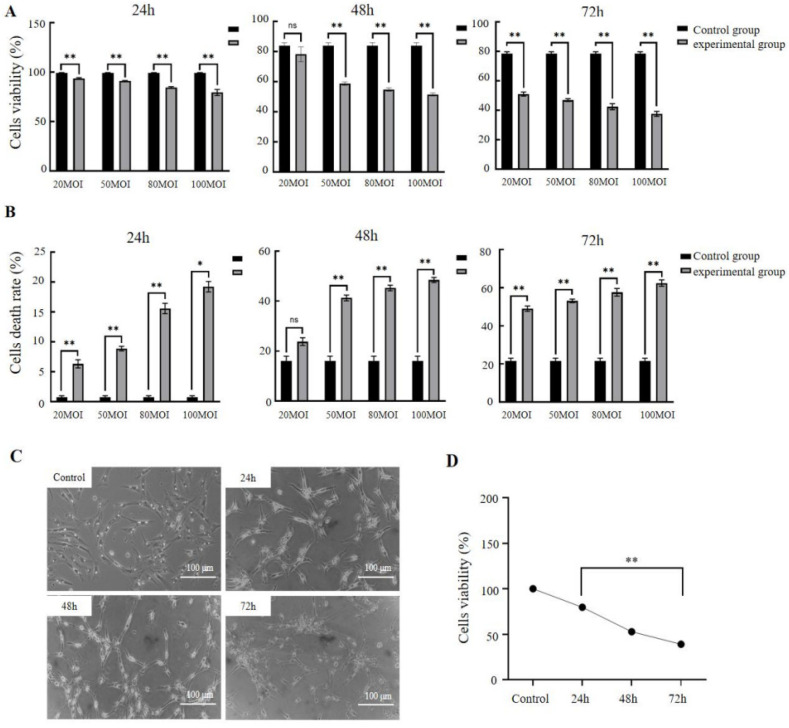
MS infection reduced the viability of chicken oviduct cells. (**A**,**B**) Cell viability assayed using the CCK-8 assay showed that MS infection inhibited chicken oviduct cells’ growth in a time and dose-dependent manner. The semi-inhibitory rate (IC50) was calculated to be 80 and 100 MOI for 48 and 72 h, respectively. (**C**) The morphology of chicken oviduct cells after MS infection under microscope. (**D**) MS infection reduced the viability of chicken oviduct cells. Data were analyzed by SPSS and cell experiments were all repeated three times. ns, no significant difference. * *p* < 0.05, ** *p* < 0.01 (Student’s *t*-test).

To investigate the biological effect of MS infection on cell proliferation, chicken oviduct cells co-culture with MS of different MOIs of 20, 50, 80 and 100 for 24 h, 48 h and 72 h, respectively, were collected for the evaluation of viability rate using a CCK-8 assay. As shown in Figure 3, MS infection reduced the chicken oviduct cell viability rate in a dose-dependent manner (Figure 3A,B). MS infection exceeding an MOI of 80 could reduce viability to below 50% after 48 h. The half-inhibitory concentrations (IC50s) of MS infection for 48 h and 72 h were 80 and 20 MOI in chicken oviduct cells, respectively. Chicken oviduct cells infected with MS at 80 MOI can cause significant morphological changes and cell death with the extension of infection time (Figure 3B,C). These results suggested that MS infection significantly inhibits chicken oviduct cell growth.

### 3.4. MS Infection Induces Apoptosis in Chicken Oviduct Cells

**Figure 4:**
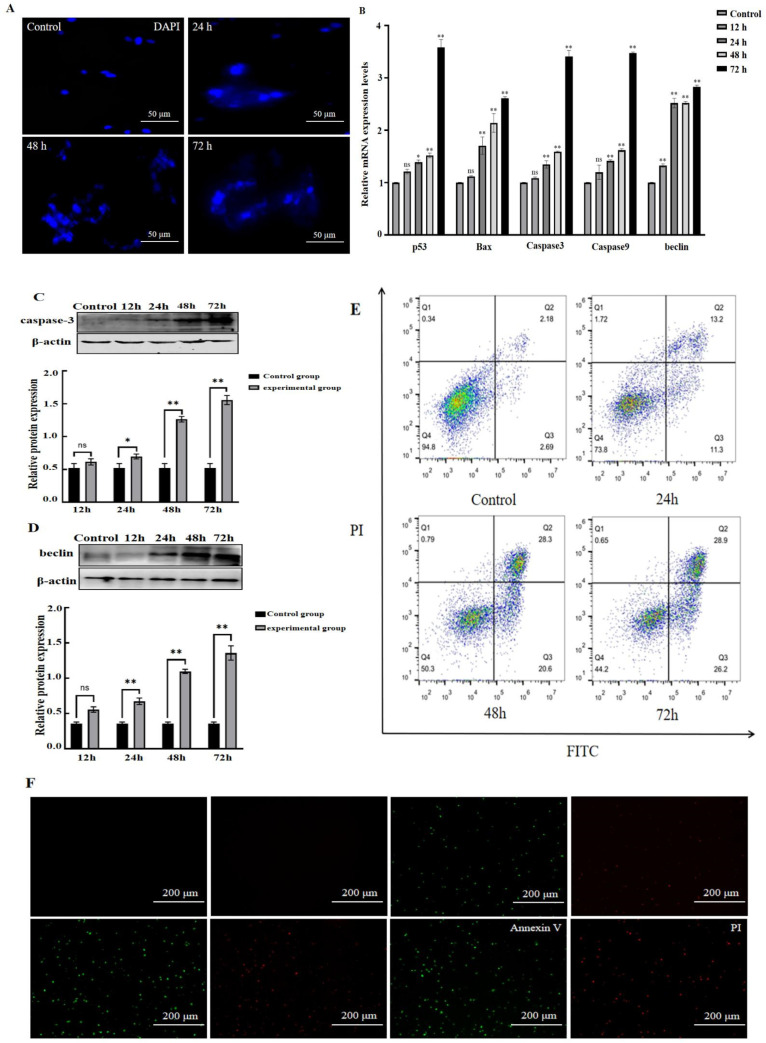
MS infection induced apoptosis in chicken oviduct cells. (**A**) Morphological changes in cell nucleus after 24, 48 and 72 h of MS infection. (**B**) MS infection increased apoptotic genes expression including p53, Beclin, Bax, Caspase 3 and Caspase 9 at mRNA levels. (**C**) Immunoblot assay showed that MS infection increased apoptosis marker Caspase-3 protein expression after 24 h. Immunoblot grayscale analysis was performed with IMAGEJ software (ImageJ-win64). (**D**) Immunoblot assay showed that MS infection increased apoptosis marker Beclin protein expression after 24 h. Immunoblot grayscale analysis was performed with IMAGEJ software. (**E**) Flow cytometry experiments showed that MS infection of chicken oviduct cells could significantly apoptotic proportion. (**F**) Annexin V-FITC/PI dual staining for cells after MS infection. Data were analyzed by SPSS, and cell experiments were all repeated three times. ns, no significant difference. * *p* < 0.05, ** *p* < 0.01 (Student’s *t*-test).

To further explore the effect of MS on chicken oviduct cells, this study detected the apoptotic indicators in chicken oviduct cells after MS infection. As shown in Figure 4, MS infection induced morphological changes in the cell nucleus. The cells’ nuclei shrink after 24 h of MS infection and fragment after 48 h (Figure 4A). Moreover, MS infection significantly increased apoptotic pathway marker genes expression, including p53, Bax, Caspase 3, Caspase 9 and Beclin (Figure 4B). Consistently, the protein levels of caspase 3 and Beclin also significantly increased after MS infection (Figure 4C,D), demonstrating that MS infection stimulated the apoptotic signaling pathway in chicken oviduct cells.

To further validate the MS-induced apoptosis effect in chicken oviduct cells, flow cytometry was performed to detecting apoptosis using Annexin V and propidium iodide (PI) dual staining. As shown in (Figure 4E,F), the proportion of apoptotic cells significantly increased after MS infection for 48 h and 72 h compared to control. Consistently, the green fluorescence representative of cell membrane damage and red fluorescence representative of nuclei exposure were observed in MS-infected cells. All these results demonstrated that MS infection induces apoptosis in chicken oviduct cells.

## 4. Discussion

As an important avian pathogen, MS can cause chronic respiratory disease, eggshell apex abnormalities, infectious synovitis, and arthritis in avian species, leading to serious economic losses in the global poultry industry [16,17]. Many studies have attempted to reveal the pathogenic mechanisms and treatment strategies of mycoplasma infection [2,3,14,18,19]. However, it is still impossible to accurately depict the specific infection process and pathogenic mechanism of MS. Additionally, MS can be transmitted horizontally and vertically [20]. Mycoplasma is mainly transmitted horizontally through the respiratory tract and colonizes the upper respiratory tract of chickens after infection, which is a critical step for successful infection [16,21]. Since MS has shown resistance to existing antibiotics, although specific subunit and inactivated vaccines have been used to prevent MS, the development of an effective vaccine remains a major challenge [8]. Understanding the pathogenesis and infection mechanism of MS can provide a basis for the prevention and treatment of MS.

MS is considered the most important avian mycoplasma from an economic and clinical point of view due to the control of antibiotics, and the fact that MS has shown resistance to existing antibiotics makes the treatment of MS difficult. Continued research on MS pathogenicity and associated virulence genes is critical, and gene sequencing, in vivo and in vitro experimental studies, whole- and partial-genome analyses, and vaccine development may contribute to the rapid detection of MS and lay the groundwork for the prevention of MS [22].

Numerous studies have demonstrated that mycoplasma can adhere to host cells through some adhesion proteins in the membrane, and mycoplasma can produce nuclease and excrete metabolites, such as hydrogen peroxide and O^2−^ into the cells through microtubules, which will destroy the activity of intracellular enzymes, cause severe damage to host cells, and accelerate host cell apoptosis [23]. It is speculated that MS may promote apoptosis in chicken oviduct cells through the action of metabolites and nucleases. However, further research is still needed to uncover the underlying mechanisms.

Our study established the MS-infected chicken oviduct cell model and confirmed that MS infection significantly inhibited the growth of chicken oviduct cells in a dose-dependent tolerance manner and caused the nuclei to crumple and fragment. Further mechanism research indicated that MS induces apoptosis by activating caspase-mediated signal pathways.

The cell death that occurs under physiological conditions takes place mainly through apoptosis, which is a non-inflammatory or silent process, whereas pathogen infection induces necrotic apoptosis or pyroptosis, which activates the immune system and causes inflammation [24,25,26]. Beclin is a dual regulator for both autophagy and apoptosis [27]. Caspase-3 is a key protein in cellular pyroptosis and apoptosis, and it also plays a critical role in regulating the growth and homeostatic maintenance of normal and malignant cells and tissues in multicellular organisms [28,29]. This study found that MS infection increased the expression of pro-apoptotic genes Caspase-3 and Beclin in chicken oviduct cells. Flow cytometry and Annexin V-FITC/PI double-staining [30] further confirmed that apoptosis occurred in MS-infected chicken oviduct cells. In fact, studies have shown that many pathogenic bacterial infections can induce cell apoptosis, including different strains of mycoplasma [1,14]. More work is still needed to figure out how to induce apoptosis and the components that induce apoptosis, which would be help to develop prevention and treatment strategies.

## Figures and Tables

**Table 1:** Primer sequences.

Genes	Primer Sequences (5′ → 3′)	Product Size
16sRNA	F: AGTAACCGATCCGCTTAAT	369 bp
	R: TACTATTAGCAGCTAGTGC	

**Table 2:** Primer sequences.

Genes	Primer Sequences (5′ → 3′)	Gene Accession Number
GAPDH	F: AAGTCGGAGTCAACGGATTT	NC_052532.1
	R: CCTTGAAGTGTCCGTGTGTA	
Bax	F: GCTCTGGTCCTCAGAAAGGG	NC_052565.1
	R: TACATCTTCCCCTGAGCGTG	
P53	F: CCAGTCACCCCTGAGACAAC	NC_052534.1
	R: ACCTCTCTGCATCCCAAAGC	
Caspase-3	F: GCTCTGGTCCTCAGAAAGGG	NC_052535.1
	R: TACATCTTCCCCTGAGCGTG	
Beclin	F: GTTGGAGACGCGACGGTTC	NC_052558.1
	R: CCACGTAGGATGGCGTGATG	

## Data Availability

The original contributions presented in the study are included in the article/Supplementary Material, further inquiries can be directed to the corresponding author.

## Supplementary Materials

The following supporting information can be downloaded at: https://www.mdpi.com/article/10.3390/vetsci11120639/s1.
