# Supplementary figures and images for: Mycoplasma synoviae Induces Apoptosis in Chicken Oviduct Cells
Source: Vet Sci. 2024 Dec 10;11(12):639. doi: 10.3390/vetsci11120639 (PMC11680356; doi:10.3390/vetsci11120639)

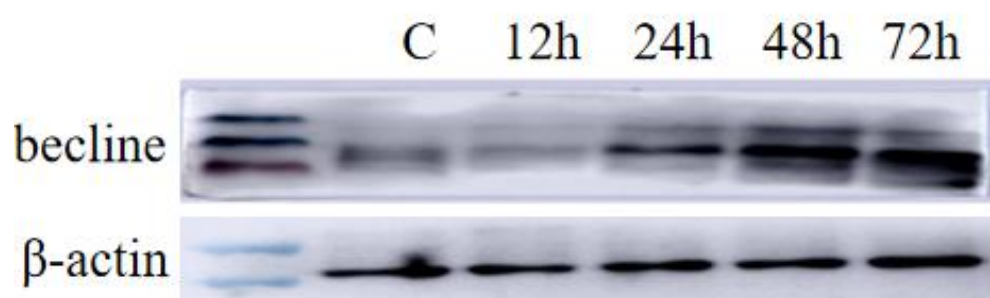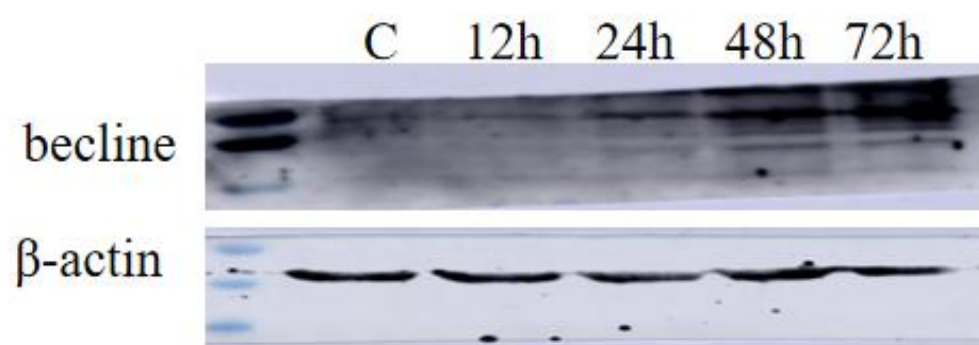

Supplement: Supplementary file 1 [file vetsci-11-00639-s001.zip › vetsci-3241351-supplementary.pdf]
